# Comparison of the Working Memory Load in *N*-Back and Working Memory Span Tasks by Means of EEG Frequency Band Power and P300 Amplitude

**DOI:** 10.3389/fnhum.2017.00006

**Published:** 2017-01-25

**Authors:** Christian Scharinger, Alexander Soutschek, Torsten Schubert, Peter Gerjets

**Affiliations:** ^1^Knowledge Media Research CenterTübingen, Germany; ^2^Laboratory for Social and Neural Systems Research, University of ZurichZurich, Switzerland; ^3^Department of Psychology, Humboldt-Universität zu BerlinBerlin, Germany; ^4^Department of Psychology, Martin-Luther-Universität Halle-WittenbergHalle, Germany; ^5^Department of Psychology, Eberhard-Karls-Universität TübingenTübingen, Germany

**Keywords:** EEG time-frequency analysis, ERD/ERS, EEG/ERP, P300, working memory, *N*-back task, operation span task, digit span task

## Abstract

According to theoretical accounts, both, *N*-back and complex span tasks mainly require working memory (WM) processing. In contrast, simple span tasks conceptually mainly require WM storage. Thus, conceptually, an *N*-back task and a complex span task share more commonalities as compared to a simple span task. In the current study, we compared an *N*-back task, a complex operation span task (Ospan), and a simple digit span task (Dspan) by means of typical WM load-related measures of the Electroencephalogram (EEG) like the parietal alpha and beta frequency band power, the frontal theta frequency band power, and the P300 amplitude, to examine whether these tasks would show commonalities or differences in WM processing-load. We expected that increasing WM-load would generally lead to a decreased alpha and beta frequency band power, an increased theta frequency band power, and a decreased P300 amplitude. Yet, based on the conceptual considerations, we hypothesized that the outcomes of these measures would be more comparable between the *N*-back and the Ospan as compared to the Dspan. Our hypotheses were partly confirmed. The *N*-back and the Ospan showed timely more prolonged alpha frequency band power effects as compared to the Dspan. This might indicate higher demands on WM processing in the former two tasks. The theta frequency band power and the P300 amplitude were most pronounced in the *N*-back task as compared to both span tasks. This might indicate specific demands on cognitive control in the *N*-back task. Additionally, we observed that behavioral performance measures correlated with changes in EEG alpha power of the *N*-back and the Ospan, yet not of the Dspan. Taken together, the hypothesized conceptual commonalities between the *N*-back task and the Ospan (and, for the Dspan, differences) were only partly confirmed by the electrophysiological WM load-related measures, indicating a potential need for reconsidering the theoretical accounts on WM tasks and the value of a closer link to electrophysiological research herein.

## Introduction

Each cognitive task of our daily live like checking a receipt by means of mental arithmetic induces load on working memory (WM). WM is defined as a cognitive construct of limited capacity where information from perception and long-term memory is temporarily maintained and manipulated (Cowan, 1999; Baddeley, 2003). WM might be fractionated into short-term memory (STM) storage components and a processing component, the central executive (Engle, 2002; Baddeley, 2003, 2007). The central executive may be fractionated further into executive functions (EF) like updating, inhibition, and shifting: the updating of information temporarily memorized for processing, the shifting of the attentional focus between different task demands, and the inhibition (or interference control) of information not (or no longer) relevant for the current processing step (Miyake et al., 2000; Baddeley, 2007; Bledowski et al., 2010). Thus, in sum, WM-load can be differentiated into WM storage-load (i.e. demanding STM processes) and WM processing-load (i.e., demanding EFs). A typical task to induce WM storage-load is the simple digit span (Dspan) task (i.e., the short-term memorization of a sequence of digits like a telephone number for later recall in correct serial order). In contrast, complex span tasks as well as *N*-back tasks are conceptualized to induce WM processing-load (see “Conceptual Task Analyses of WM Span Tasks and *N*-Back Tasks” for detailed task analyses).

Whereas, *N*-back tasks have been extensively studied in brain-imaging (e.g., Jonides et al., 1997; Owen et al., 2005) and electrophysiological research (e.g., Gevins and Smith, 2000; Krause et al., 2000; Pesonen et al., 2007; Palomäki et al., 2012; Scharinger et al., 2015), complex span tasks like the reading span or the operation span (Ospan) task (Daneman and Carpenter, 1980; Turner and Engle, 1989), that originate from individual differences research of cognitive psychology (e.g., Bayliss et al., 2003; Unsworth and Engle, 2007), have not been examined using neurophysiological measures like the Electroencephalogram (EEG). Thus, it remains an open question whether *N*-back and complex span tasks – despite all being conceptualized as WM tasks that require WM processing – would show common or dissociable WM load-related EEG correlates that might be different from the simple Dspan task (i.e., a task that mainly requires WM storage).

The current study addressed this issue by investigating typical EEG measures for WM-load like the EEG theta (4–6 Hz), alpha (8–13 Hz), and beta (14–24 Hz) frequency band power (Gevins and Smith, 2000; Pesonen et al., 2007; Palomäki et al., 2012; Scharinger et al., 2015), and the P300 amplitude (Wickens et al., 1983; Watter et al., 2001; Scharinger et al., 2015) for an Ospan, an *N*-back, and a Dspan task. Our research is further motivated as comparisons between *N*-back and WM span tasks to date have been solely done by means of behavioral data (Kane et al., 2007; Miller et al., 2009; Jaeggi et al., 2010). These studies often show surprisingly weak correlations between *N*-back and complex span tasks, given the conceptualization of both task families as WM tasks (for a recent review and meta-analysis, see Redick and Lindsey, 2013).

In the following, we will briefly provide conceptual task analyses of WM span and *N*-back tasks highlighting common or dissociable demands on WM processing raised therein. Then, we will describe typical EEG measures of WM-load, and finally, summarize our hypotheses with respect to the expected outcomes of these measures on the different tasks.

### Conceptual Task Analyses of WM Span Tasks and N-Back Tasks

Complex span tasks are dual-tasks consisting of a processing subtask and a STM subtask. For example, in a classical Ospan task (Turner and Engle, 1989), participants have to validate the results of simple equations (e.g., 3 × 5 - 10 = 4?) intermixed with the memorization of certain items (e.g., words, letters, or digits). After several trials, each consisting of an equation-validation paired with a memory-item, subjects have to recall the memorized items in correct serial order. Because of the dual-task character, complex span tasks put severe demands on WM processing and thus on EFs: Apart from the updating of WM content, subjects have to shift between the two subtasks and to inhibit currently irrelevant information. These demands on EFs might especially be raised at the beginning of each memorization subtask within the complex span task, that is, when participants have to shift from the processing subtask to the memorization subtask that requires WM updating as well as the inhibition of the previous task set and of incorrect response tendencies.

Simple span tasks (Humstone, 1919; Blankenship, 1938; Gregoire and Van Der Linden, 2004) consist merely of the memorization of a sequence of items (e.g., digits in the case of a Dspan task) for later recall in correct serial order. These tasks do not incorporate any additional processing components. Thus, in contrast to complex span, simple span tasks are supposed to mainly demand STM storage rather than WM processing.

In *N*-back tasks (e.g., Jonides et al., 1997; Gevins and Smith, 2000; Krause et al., 2000; Owen et al., 2005; Pesonen et al., 2007; Palomäki et al., 2012; Scharinger et al., 2015) participants have to compare a current stimulus with a stimulus they saw *N*-steps back in the sequence. Thus, for increasing *N*, the *N*-back task increasingly demands WM processing. In the 1-back task condition subjects have to continuously update the stimuli maintained in WM. In the 2-back and above, subjects additionally have to shift the attentional focus between the stimuli for comparison and inhibit stimuli no longer needed to be maintained in WM as well as incorrect response tendencies for stimuli at the wrong position in the sequence (Jonides et al., 1997; Chen et al., 2008). Due to the number of processes involved, the *N*-back task may have a dual-task character (Watter et al., 2001).

In sum, conceptually both, *N*-back and complex span tasks comparably require WM processing (i.e., demand the EFs updating, shifting, and inhibition). In contrast, simple span tasks mainly induce WM storage-load and less WM processing-load (i.e., only demanding the EF updating). Importantly, in the *N*-back task the demands on the EF updating, shifting, and inhibition are closely intertwined as participants have to perform stimuli comparison and memorization processes in the same temporal window resulting in simultaneous demands on EFs. In complex span tasks, the demands on EFs might be more sequential: Subjects first perform the processing sub-task and then shift to the memorization sub-task. Thus with respect to the WM load-related measures of the EEG, we expected to observe commonalities but also differences between all three tasks.

### EEG Frequency Band Power Correlates of WM Processing-Load

Time-frequency representations (TFRs) plot oscillatory EEG activity as a function of frequency band power values. Using TFRs, several studies examined the load-related changes in the EEG theta, alpha, and beta frequency bands at frontal and parietal recording sites for *N*-back tasks (Pesonen et al., 2007; Krause et al., 2010; Palomäki et al., 2012; Scharinger et al., 2015). For increasing *N*-back levels, these studies concurringly report decreased alpha and beta frequency band power at parietal electrodes (i.e., the so-called event-related desynchronization, ERD) that is often accompanied by increased theta power at frontal electrodes (i.e., the so-called event-related synchronization, ERS; Gevins et al., 1997; Gevins and Smith, 2000). Furthermore, Pesonen et al. (2007) observed that the alpha ERD was not only more pronounced in terms of absolute values but also in terms of longer durations for the higher *N*-back load levels (2-back, 3-back) as compared to lower load levels (1-back). EEG alpha effects especially might reflect demands on WM processing (Klimesch, 1999). Theta effects might especially reflect cognitive control functions in WM (Sauseng et al., 2010). Taken together, theta, alpha, and beta frequency band power proved to be sensitive to WM processing in the *N*-back task.

To the best of our knowledge, EEG frequency band power correlates of complex span tasks have not been studied before. One study using a simple Dspan task (Stipacek et al., 2003) reported a decreased mean alpha power for increased numbers of to-be-remembered digits, that is for increased STM storage demands (contrary to these findings, however, other studies reported increased alpha power for higher STM storage demands, e.g., Jensen et al., 2002; Palva and Palva, 2007). Thus, one specific research goal of the current study was to systematically analyze and describe EEG frequency band power TFRs for span WM tasks for the first time, and to assess whether these tasks induce comparable or dissociating WM processing-load indicators as the *N*-back task.

### EEG P300 Amplitude Signaling WM Processing Demands

Besides frequency band power, the analyses of event-related potentials (ERPs) like the P300 provide insights into the neuronal activity underlying WM performance. The P300 typically shows a maximal positive deflection in a time range between 250 and 500 ms post-stimulus onset at parietal electrodes (Johnson, 1993; i.e., the P3b; Polich, 2007). Traditionally, the P300 has been observed to be elicited for deviant stimuli amongst standard stimuli in oddball paradigms (Squires et al., 1975), when in a sequence of stimuli (i.e., the standards) some rare stimuli (i.e., the deviants) are presented that differ from the standards with respect to certain stimulus dimensions. Amongst others, the P300 amplitude has been shown to reflect the predictability of deviants (e.g., Picton, 1992), their physical deviance from the standards, their subjective categorization, relevance judgment, and attention to (Sutton et al., 1965; Ruchkin and Sutton, 1978; Picton, 1992; Gray et al., 2004). In sum, the P300 reflects processes of attention allocation and memory updating (Picton, 1992; Polich, 2007) and, more specifically (but controversially discussed), might index the updating of an established context in mind (Donchin and Coles, 1988; Verleger, 1988).

In *N*-back tasks, the P300 amplitude decreases for increasing WM-load (McEvoy et al., 2001; Watter et al., 2001; Chen et al., 2008; Scharinger et al., 2015), thus showing a comparable outcome as in dual-task studies (e.g., Wickens et al., 1983; Kok, 2001; Fu and Parasuraman, 2008). This observation led Watter et al. (2001) to conclude that the *N*-back task might incorporate some dual-task nature. In general, according to these authors, the decreasing P300 amplitude serves as an index for the internal distribution of controlled attention when different (executive) WM functions are required.

In simple span tasks, only few studies have studied the P300, yielding mixed results. Grune et al. (1996) studied the P300 amplitude in a Dspan task. Participants had to remember sequences of seven digits, each digit presented one after another on the screen. The authors compared the averaged ERP curves for each digit and found a decrease of the P300 amplitude for increasing numbers of to-be-remembered digits. However, a more detailed examination of the results revealed a significant decrease of the P300 amplitude only up to remembering four digits. Contrary to these results, a study by Gross et al. (1992) found an increased (instead of a decreased) P300 amplitude for increased numbers of to-be remembered digits.

In sum, the results concerning the P300 amplitude in span tasks are sparse and conflicting for simple span tasks like the Dspan task, and, to the best of our knowledge, are lacking for complex span tasks like the Ospan task. Given previous results of factors that trigger the elicitation of a P300 and factors that modify the P300 amplitude, we would expect to observe a P300 in both, the *N*-back and the complex span task showing a decreased amplitude for increased WM-load. This is because both tasks require context updating and both tasks might share some dual-task characteristics (Watter et al., 2001). In contrast, a simple span task might elicit a less pronounced P300 that lacks the decrease for increased WM-load.

### The Current Study

To sum up, in the current study we were interested in examining typical EEG correlates of WM processing-load like the EEG theta, alpha, and beta frequency band power and the P300 amplitude. We analyzed these measures for a numerical *N*-back task, the memorization subtask of an Ospan and the memorization phase of a Dspan task. Noteworthy, we closely matched the three tasks with respect to time constraints (i.e., timing of trials), number of trials, the overall duration, and, most important, the time-window chosen for data analyses (see Data Preprocessing and Analysis).

Because of their conceptualization as genuine WM tasks and their comparably assumed dual-task nature, we hypothesized that both, the *N*-back and the Ospan task, comparably demand WM processing (i.e., the EFs like updating, shifting, and inhibition). In contrast, we hypothesized the Dspan task to demand less WM processing (i.e., potentially mainly updating). Therefore, when comparing three load levels of low, medium, and high WM processing-load (with separately defined load levels for each task based on behavioral performance to match different task difficulties, see Data Preprocessing and Analysis), we expected to observe EEG patterns in the TFRs and the P300 being more similar for the *N*-back and the Ospan as compared to the Dspan task. In line with this reasoning, we expected to observe stronger alpha and beta ERDs (and theta ERSs) for these former two WM tasks as compared to the simple Dspan task, as these tasks conceptually should induce more WM processing-load than the simple Dspan task.

## Materials and Methods

### Participants

Twenty university students (age: *M* = 25.15, *SD* = 3.15; 11 females) participated in the study and received a payment of 8 €/h. They were all native speakers of German, right-handed according to the Edinburgh Handedness Inventory (Oldfield, 1971) and reported no neurological disorders. All participants had normal or corrected-to-normal visual acuity. The study was approved by the local ethic committee of the Knowledge Media Research Center, Tübingen. Participants gave their written consent at the beginning of the study.

### Materials

#### N-Back Task

Eight different single digits (1–9, except 7) served as stimuli in the *N*-back (the 7 was excluded because in contrast to all other used digits it consists of two syllables when verbally encoded, i.e., ‘seven’; thus potentially it may load verbal WM differently). The digits were either printed in blue (RGB-values: 51,75,177) or in yellow (255,215,0) on black background and printed in one of four different fonts (Arial; Curlz MT; Viner Hand ITC; Castellar) in 25 points font size each. This combination of two colors and four fonts resulted in eight different stimuli forms. The assignment of digit value, position, and form (color and font) was done randomly for the current stimuli in the *N*-back sequences.

The stimuli were presented at one of the eight outer positions of a 3×3 grid. The grid was centrally located on the screen and was marked through gray colored thin horizontal and vertical lines. A fixation cross marked the middle of the grid. The height and width of the grid was about 5.5 cm each. Participants were instructed to always keep the central fixation cross fixated. The *N*-back stimuli thus occurred within a visual angle of maximally about 4° horizontally and about 4.5° vertically, that is, the stimuli were within participants’ visual field and did not require eye movements.

**FIGURE 1:**
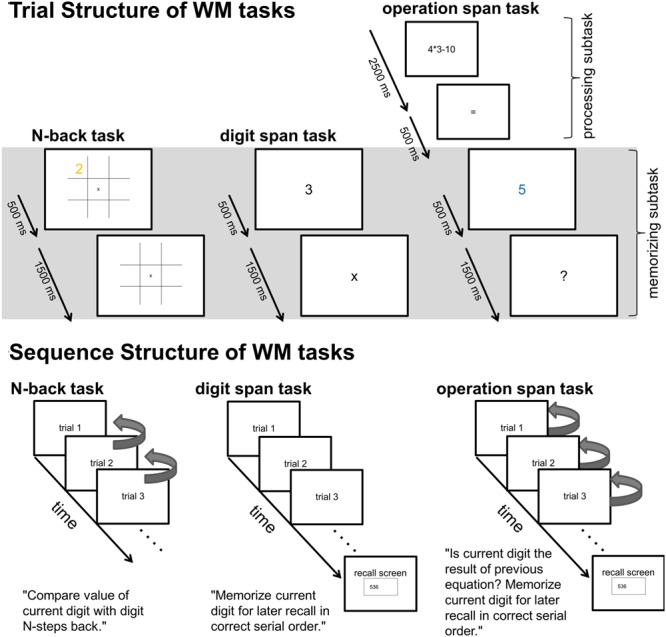
**Upper part: Trial structure of single *N*-back, Dspan, and Ospan trials.** The gray shaded area indicates the time-windows (2000 ms long for all tasks) of the EEG data analyses. Lower part: Schematic sequence of the three tasks. The arrows on the right side of the *N*-back trials exemplarily indicate the comparisons subjects had to make within a 1-back sequence (i.e., for each trial, subjects had to compare the current digit-value with the previous digit-value, signaling a match or mismatch via key-press). The arrows on the right side of the Ospan trials indicate the comparisons subjects had to make between the current digit and the result of the previous equation (i.e., within each trial, subject had to compare the current digit with the result of their previous mental calculation, signaling a match or mismatch via key-press).

The stimuli were presented sequentially for 500 ms followed by 1500 ms of black screen with only the gray grid lines and the central fixation cross visible (cf. **Figure 1**). In sum one trial lasted 2000 ms. A sequence of 34 trials constituted an *N*-back block. The first four trials of a block were excluded from data analysis. The total duration of a block (including task instruction) summed up to about 90 s. During an *N*-back block, participants had to indicate via key-press whether, or not, the stimulus of the current trial matched the stimulus they saw *N*-steps back with respect to a certain stimulus dimension (i.e., digit value, location, or form). One third of the trials of a block were *matches*, that is, required participants to press the ‘yes’-key (‘m’) as correct response, two third of the trials were *mismatches*, that is, required participants to press the ‘no’-key (‘x’) as correct response. The sequences of matches and mismatches were pseudo-randomly generated with the constraint that after a maximum of three matches at least one mismatch followed.

At the beginning of each *N*-back block the current task condition and the *N*-back level was announced. We used four *N*-back levels (1-back, 2-back, 3-back, 4-back) and three task conditions (digit value, position, form), albeit data analyses for comparison of the *N*-back and the span tasks was restricted to the numerical (digit value) *N*-back condition^1^. During blocks of the task condition ‘digit value,’ participants were instructed to focus only on the digit values and to perform the *N*-back comparison process only for the digit stimulus dimension and ignore the other two dimensions (position and form). During blocks of the task condition ‘position,’ participants had to focus only on the position of the stimuli while ignoring digit values and forms, during blocks of the task condition ‘form’ they had to focus only on the form (color and font) of the stimuli while ignoring the other two dimensions.

In total, we used 12 different task conditions (digit value, position, and form, each 1-back to 4-back), each assigned to three blocks. This resulted in 36 blocks in total. The sequence of the 36 blocks was randomly created for each participant. Importantly, all blocks were perceptually identical. Only the pre-block task instruction defined the task at hand for the participants. Participants performed a training session for each *N*-back level and *N*-back condition (with blocks of only 20 trials) once before the actual task (36 blocks) started. The total duration of the *N*-back tasks, including training and breaks, summed up to about 90 min.

#### Dspan Task

We used eight different single digits (1–9, except 7) as stimuli for the Dspan task (i.e., the same digits as in the *N*-back). The digits were printed in gray color on black background in Arial font (25 points font size). Sequences of single digits were presented at the center of the screen. Each digit was shown for 500 ms, followed by a fixation-cross for 1500 ms. Thus, a Dspan trial was of the same length as an *N*-back trial. Participants were instructed to remember the digits they saw on the screen in correct order. After three to eight trials (digits) a recall screen was shown where participants had to type in the remembered digits. Participants were allowed to take a self-paced short break after having typed in the digits. The next Dspan sequence started when participants pressed the ‘return’-key.

Participants did not know beforehand the length of the current Dspan sequence (i.e., at which trial position the recall-screen would occur). We used 30 sequences of eight digits, and 18 sequences of seven to three digits each, respectively. At the end of these sequences the recall screen was shown. The single digits of a sequence as well as the order of the sequences were randomly chosen for each participant. Importantly, as the higher Dspan sequences incorporated trials of the lower sequences (e.g., in a Dspan sequence of eight digits are incorporated trials of the lower sequences one to seven), the total amount of trials per load level (i.e., the current trial position in a Dspan sequence) was additive by the factor 18. Thus, we had 30 trials of load level eight, 48 trials of load level seven, 66 trials of load level six and so on. The duration of a sequence varied between 6 and 16 s, depending on length of the sequence. The total duration of the Dspan task (including the recall screens) summed up to about 35 min. Therefore, we split the Dspan into two parts of about 17 min length that were presented in alternation with two parts of the Ospan task (cf. below).

#### Ospan Task

The Ospan task design built upon the Dspan task design: Participants had to remember sequences of single digits (1–9, except 7) of different length (memorization subtask). Additionally, they had to perform simple calculations within each trial (processing subtask). Each trial in the Ospan task started with a simple equation (e.g., 3 × 4 - 8), centrally presented on the screen (Arial, 25 points font size, gray-colored, black background). The equation was shown for 2500 ms followed by an equal sign presented for 500 ms. Then a single digit (1–9, except 7) was shown for 500 ms as possible result of the equation. This digit was presented in blue to ease participants’ comprehension of the task instruction, which digit they had to remember for later recall (see below). After a question mark, presented for 1500 ms, the next trial began. The total duration of an Ospan trial summed up to 5 s.

The task was to indicate via key-press whether the single digit presented before the question mark was the correct result of the preceding equation or not. The equation was either of the form of a multiplication combined with a subtraction (a × b - c) or a division combined with an addition (c/b + a), with ‘a’ and ‘b’ being single digits (1–9, except 7) and ‘c’ being a two digits number. The third operator, ‘c,’ was chosen to create a result of the equation that again was a one digit number (1–9, except 7). Assignment of response keys and length of the response window was the same as in the *N*-back task, as well as the probability of 1/3 matches (i.e., correct result shown) and 2/3 mismatches (i.e., wrong result shown). Additionally, like in the Dspan task, participants had to remember these (colored) digits (i.e., the possible results) for later recall in correct order. After sequences of three to seven trials, a recall screen was shown, identical to the procedure of the Dspan task described above.

For the Ospan task, we set the maximal length of a sequence to seven trials. We had 30 sequences of length seven, and like in the Dspan task, 18 sequences of length six to three, respectively. Thus, the amount of trials per load level was comparable to the Dspan with 30 trials for the load level seven, 48 trials for the load six, 66 trials for the load five, and so on. The duration of a sequence varied between 15 and 35 s, depending on length (three to seven trials). The total duration of the Ospan task (including the recall screens) summed up to about 55 min. We split the Ospan task into two parts of about 28 min length that were presented in alternation with two parts of the Dspan task (cf. Dspan Task).

### Procedure

Participants performed two experimental sessions on two different days within 1 week. Each session, including EEG preparation and breaks, lasted to about 2.5 h. In the first session, participants performed the *N*-back tasks. In the second session, the same participants performed the Ospan and Dspan task, each split into two parts and in alternation with one another. The order of the span tasks was counterbalanced across participants. At the beginning of each session, participants performed training trials of the sessions’ tasks.

### Apparatus

The study was run in a quiet room that was dimly lightened. Participants sat in a comfortable chair in front of a 17-inch monitor (iiyama ProLite E481S, 1024 × 768 pixels screen resolution, about 70 cm viewing distance) while their EEG data were recorded. EEG data were recorded from 27 electrode sites (Fp1, Fp2, F7, F3, Fz, F4, F8, FC5, FC1, FC2, FC6, T7, C3, Cz, C4, T8, CP5, CP1, CP2, CP6, P7, P3, Pz, P4, P8, O1, O2) positioned according to the international 10/20 system (Jasper, 1958). The right mastoid served as reference during recording. Ground electrode was positioned at FPz. Three additional electrodes were placed around the eyes for EOG recording. EEG data were recorded with the BCI 2000 toolbox (Schalk et al., 2004) at 512 Hz sampling rate (two 16 channels g.USBamp Generation 3.0 amplifiers, g.tec medical engineering, Inc.) using active electrodes (ActiCap, Brainproducts, Inc.). For technical reasons, the EEG data of four participants were recorded using a different amplifier (ActiCHamp, Brainproducts, Inc.) and recording software (PyCorder 1.0.2). Impedances were kept below 5 kΩ.

### Data Preprocessing and Analysis

Electroencephalogram data were preprocessed and analyzed using customized analysis scripts (Matlab 2012b, MathWorks, Inc.; EEGLAB v. 11.0.5.4b; Delorme and Makeig, 2004). During preprocessing the continuous EEG data were filtered (low-pass 40 Hz, high-pass 0.5 Hz, linear finite impulse response filters). Electro-Occulogram (EOG) artifacts were corrected using independent component analysis (ICA) decompositions. Independent components (ICs) identified as EOG-ICs by visual inspection were rejected. EEG data were re-referenced to average reference.

After preprocessing, the continuous EEG data were divided into stimulus-locked epochs of 2000 ms length. We chose a data analysis window that covered aspects of the three different tasks that were highly comparable (see **Figure 1**). Within the data analysis window in all tasks participants were presented digits (500 ms) they had to remember followed by a 1500 ms retention period. In the *N*-back task and the Ospan task participants additionally had to perform decision processes during this 2000 ms time-window.

An automatic artifact removal was performed with respect to the EEG data: Epochs that exceeded ±80 μV were excluded from further analyses (Duncan et al., 2009). Using this criterion and a subsequently following visual inspection of the EEG data, epochs containing severe artifacts (e.g., muscle artifacts) were excluded from any further data analysis. No further artifact removal or correction was performed on the EEG data. On average, 2.88% (*SD* = 5.28) of all trials had to be excluded due to these criteria. With respect to the *N*-back task, only trials that the participants correctly responded to were analyzed further. In the final data set for all tasks, the minimal trial number per condition was above 50 artifact-free data epochs, in line with recommendations for conducting P300 data analysis (e.g., Duncan et al., 2009).

**FIGURE 2:**
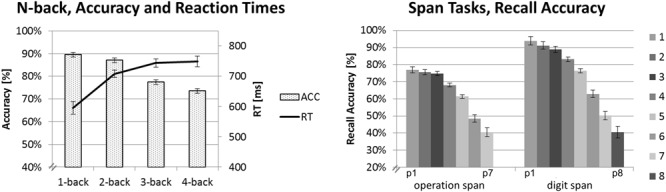
**Behavioral data results.** Mean accuracy and RTs for the *N*-back task on the left-hand side. Mean recall accuracy for each digit position for the Ospan (positions p1 to p7) and the Dspan task (positions p1 to p8). Black error bars indicate ±1 standard error of the mean.

For the span tasks, we calculated the recall accuracy for the different digit positions in the sequence (p1 to p7 and p8, respectively), that is, the recall accuracy for increasing WM-load. The recall accuracy of a digit trial in the sequence was calculated as the percentage of correctly recalled digits at that specific digit position with respect to the total amount of trials of this specific digit position (i.e., we performed the first step of the partial-credit, unit scoring procedure used for the calculation of an overall span score as described by Conway et al., 2005). We run a one-factorial repeated-measures ANOVA and *post hoc* pairwise comparisons (paired *t*-tests, two-tailed) on these accuracy values. This analysis (see “Ospan Task” and “Dspan Task”) revealed certain steps within the digit sequence where recall accuracy significantly dropped and others parts within the sequence for which recall accuracy remained quite stable (see **Figure 2**). For example, the accuracy was quite comparable for remembering one to three digits and it was significantly reduced for remembering four digits. Based on this analysis, we defined three load-categories of *low, medium*, and *high-load*. In the Ospan the low-load category consisted of trials of the third digit to be remembered (i.e., p3) within the sequence of trials of the memorizing subtask. The medium-load category consisted of trials of the fifth digit (i.e., p5) and the high-load category consisted of combined trials of the sixth and seventh digit to be remembered (i.e., p6 + p7). In the Dspan task, the low-load category consisted of trials of the fourth digit to be remembered (i.e., p4), the medium-load category consisted of trials of the sixth digit (i.e., p6), and the high-load category consisted of combined trials of the seventh and eighth digit to be remembered (i.e., p7 + p8). We used a different load assignment of these three categories in the Ospan and the Dspan to approximately match the difficulty levels between the span tasks as indicated by the recall accuracy. Note that we combined trials of two digit positions for the high-load conditions to increase the total number of trials of these conditions which would have otherwise been rather low for a concise EEG data analysis. In the *N*-back task reaction time (RT) and accuracy indicated no significant difference between 3-back and 4-back load levels. Therefore, we used the 1-back to 3-back load level as representative for defining the load levels low, medium, and high and excluded the 4-back load level from any further analysis.

#### TFRs

We calculated mean TFRs for the following regions of interest (ROIs): frontal-central (electrodes Fc1, Fz, Fc2, Cz) and parietal-occipital (P3, Pz, P4, O1, O2), for each task, load level and participant separately within a frequency band range from 2 to 32 Hz and a time range from 0 to 2000 ms. The ROIs were chosen based on literature reporting maximal effects of WM-load for the theta frequency band power at frontal-central electrodes and maximal effects of WM-load for the alpha and beta frequency band power at parietal-occipital electrodes (e.g., Gevins et al., 1997; Gevins and Smith, 2000; Pesonen et al., 2007; Krause et al., 2010). The frequency band power for the TFRs was calculated using stepwise fast-fourier transforms (FFTs, 500 ms width moving windows, 10 ms steps) applied over the entire epoch length. Then the percentages of event-related desynchronization/synchronization (ERD/ERS%; Pfurtscheller and Lopes da Silva, 1999) were calculated for each data point with respect to a baseline. As baseline we used the mean frequency band power of the low-load category (i.e., the averaged power over the entire epoch length of the low-load condition) for each task individually.

For a more comprehensive statistical analysis, we calculated the mean ERD/ERS%-values for theta (4–6 Hz), alpha (8–13 Hz), and beta (14–24 Hz) frequency band power in an early time-window (0–1000 ms post-stimulus onset) and a late time-window (1000–2000 ms post-stimulus onset). The two time-windows were chosen based on visual inspection of the TFR plots indicating generally more pronounced effects between 0 and 1000 ms post-stimulus onset (i.e., the early time-window) and, for the medium and high-load levels longer lasting alpha ERD effects up to about 1800 ms post-stimulus onset in the *N*-back and the Ospan task as compared to the Dspan task (i.e., in the late time-window). For each frequency band we ran separate three-factorial repeated-measures ANOVAs with the within-factors task (*N*-back, Dspan, Ospan), time-window (early, late), and WM-load (low, medium, high). As previous studies commonly reported maximal effects of WM-load for the theta frequency band power at frontal-central electrodes and maximal effects of WM-load for the alpha and beta frequency band power at parietal-occipital electrodes (e.g., Gevins et al., 1997; Gevins and Smith, 2000; Pesonen et al., 2007; Krause et al., 2010), we restricted our statistical analyses on the frontal-central ROI for the theta frequency band power mean ERD/ERS%-values and on the parietal-occipital ROI for the alpha and beta frequency band power ERD/ERS%-values. Greenhouse–Geyser corrections were performed on the *p*-values where necessary. For *post hoc* pairwise comparisons (*t*-tests, two-tailed) all *p*-values were Bonferroni corrected for multiple comparisons. Level of significance was set at α = 0.05 for all analyses and partial eta-square ($\eta_{p}^{2}$) is reported as a measure of effect size.

#### Correlational Data Analysis

For an exploratory correlational data analysis of the frequency band power data and the behavioral task performance^2^ we calculated overall performance measures for the *N*-back and the span tasks. The performance measure (called ‘score’ henceforth) for the *N*-back task was defined as the average accuracy over all four *N*-back levels. For the span tasks the scores were calculated by averaging the recall accuracies of all digit positions of all trials in the sequence. These behavioral performance scores were correlated (Pearsons’ correlations coefficients, two-tailed) with the mean alpha ERD, beta ERD, and theta ERS (all averaged over the two data analysis windows described above).

#### ERPs

Single trial EEG data epochs (0–1000 ms post stimulus onset) were baseline corrected using a -150 ms pre-stimulus baseline. Paired *t*-tests (two-tailed) between load levels were conducted for each time point (EEGLAB bootstrapping statistics, using false-discovery-rate corrections for multiple comparisons) at frontal (F3, Fz, F4), central (C3, Cz, C4), and parietal (P3, Pz, P4) electrodes for each task separately. These electrodes were chosen in line with literature (e.g., Watter et al., 2001). For a more comprehensive statistical analysis, we calculated the mean amplitude in a time-window between 300 and 500 ms post-stimulus onset at electrode Pz. On these data, we run a two-factorial repeated-measures ANOVA with the factors task (*N*-back, Dspan, Ospan) and WM-load (low, medium, high). We used the same statistical criteria for this ANOVA as reported above.

## Results

### Behavioral Data

For each task, we run separate one-factorial repeated-measures ANOVAs, using the same criteria as reported above for the ANOVAs on the EEG data. **Figure 2** shows the mean recall accuracies of the span tasks (for digit positions p1 to p7 and p8, respectively) and the accuracies and RTs of the *N*-back task.

#### *N*-Back Task

Reaction time was calculated only for correctly responded trials. For RT, the one-factorial repeated-measures ANOVA revealed a main effect of load, *F*(3,57) = 14.51, *p* < 0.001, $\eta_{p}^{2}$ = 0.43. The strongest increase in RT could be observed for the step from the 1-back (595 ms) to the 2-back task condition (708 ms, *p* < 0.001). As can be seen in **Figure 2**, the RT increased further on the higher load levels (3-back: 744 ms, 4-back: 748 ms), however, this increase was rather marginally and statistically not significant between load levels above the 2-back level (all *p* > 0.810).

Accuracy decreased with increasing *N*-back load as shown by a main effect of load, *F*(3,57) = 24.51, *p* < 0.001, $\eta_{p}^{2}$ = 0.56. Interestingly, accuracy did not differentiate between most of the directly adjacent load levels: 1-back (89%) and 2-back (87%, *p* = 0.737) or 3-back (77%) and 4-back (74%, *p* = 0.218). It did, however, significantly decline between the 2-back and 3-back load levels (all *p* < 0.003).

On the basis of these findings, we decided to use only the *N*-back levels 1-back to 3-back for a comparison with the three defined load-categories (i.e., low, medium, and high) of the span tasks: RT increased most between 1-back and 2-back load level, accuracies decreased significantly between 2-back and 3-back load levels. No significant difference could be observed between 3-back and 4-back load levels on neither RTs nor accuracies (all *p* > 0.218).

#### Ospan Task

In the Ospan task, we observed a significant decrease of recall accuracy with increasing digit positions, as revealed by a main effect of load, *F*(6,114) = 58.18, *p* < 0.001; $\eta_{p}^{2}$ = 0.75. *Post hoc* pairwise comparisons showed initial steps of comparable recall accuracy followed by significant drops in accuracy. Recall accuracy for trials at digit position p1 to p3 was statistically equal (p1: 77%, p2: 76%, p3: 75%, all *p* = 1.00) and significantly higher than recall accuracy for trials at digit position p4 and following (all *p* < 0.004). Starting from digit position p4 recall accuracy significantly decreased for each digit position in the sequence (p4: 68%, p5: 61%, p6: 49%, p7: 41%, all *p* < 0.019). Although recall accuracy dropped significantly between p6 and p7 (*p* = 0.014), we decided to combine these two load levels in order to create a high-load level category that contains a sufficient amount of trials for EEG data analysis (cf. Data Preprocessing and Analysis). Overall, our classification of three load-categories and the assignment of digit position p3 to the low-load category, p5 to the medium-load category and combined p6 and p7 to the high-load category as described in Section “Data Preprocessing and Analysis” seemed to be justified by the results of this statistical analysis. We have to underline again that for all tasks we defined such three individually defined load-categories (i.e., low, medium, and high-load) based on the behavioral data. This way we sought to minimize possible task differences with respect to general task difficulty and to create reasonably defined load-categories of comparable task difficulty (i.e., easy, medium, difficult) for which the EEG data then could be compared.

Additionally, we checked the performance in the processing subtask of the Ospan task (i.e., the accuracy and RT for the decision, whether the given result is the correct or incorrect result of the preceding equation). The accuracy of the processing subtask (equation-result decision) numerically decreased slowly and showed in average 75% correct responses, with a range between 78% (*SD* = 11) at trial position p1 to 70% (*SD* = 14) at trial position p7. A one-factorial repeated-measures ANOVA revealed main effect of load, *F*(6,114) = 4.66, *p* = 0.006, $\eta_{p}^{2}$ = 0.19, yet *post hoc* pairwise comparisons (*t*-test, two-tailed) did not show any significant differences (all *p* > 0.133). In turn, the RT of the equation-result decision numerically increased for increased trial positions from p1, 731 ms (*SD* = 161) to p7, 761 ms (*SD* = 206). A one-factorial repeated-measures ANOVA revealed main effect of load, *F*(6,114) = 2.85, *p* = 0.038, $\eta_{p}^{2}$ = 0.13, yet, as for accuracy, *post hoc* pairwise comparisons (*t*-test, two-tailed) did not show any significant differences (all *p* > 0.083). In sum, these results indicated that (as expected) participants were equally well performing both subtasks of the Ospan task (i.e., the processing subtask and the memorization subtask), thus confirming the successful execution of the task.

#### Dspan Task

In the Dspan task, we also observed a significant decrease of recall accuracy with increasing digit position, as revealed by a main effect of load, *F*(7,133) = 71.45, *p* < 0.001; $\eta_{p}^{2}$ = 0.79. Like in the Ospan task, we observed initial steps of comparable recall accuracy followed by significant drops in accuracy. Recall accuracy was comparable between trials at digit positions p1 and p2 (p1: 94%, p2: 91%, *p* > 0.754) as well as p2 and p3 (p3: 89%, *p* = 1.00). Recall accuracy then dropped significantly (p4: 83%, p5: 76%, p6: 63%, p7: 50%, p8: 41%, all *p* < 0.004). Based on these results, we assigned digit position p4 to the low-load category, p6 to the medium-load category, and combined p7 and p8 to the high-load category.

To sum up, the behavioral data of the *N*-back and the span tasks are in accordance with literature and confirmed that we have successfully manipulated the load in the current tasks. For increasing WM-load due to increasing *N*-back levels or to-be-remembered digits, the accuracy decreased and the RT in the *N*-back increased (e.g., Turner and Engle, 1989; Gevins and Smith, 2000).

### EEG Alpha, Beta, and Theta Frequency Band Power TFRs

**FIGURE 3:**
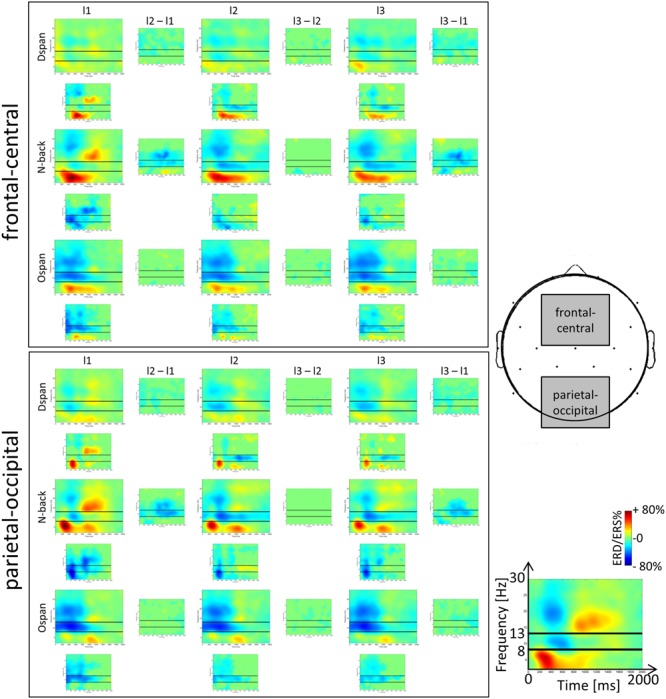
**Time-frequency representations (TFRs) of the ERD/ERS%-values of the *N*-back, Ospan and Dspan task in a frequency range from 2 to 32 Hz and a time range from 0 to 2000 ms post-stimulus onset at frontal-central (Fc1, Fc2, Fz, Cz) and parietal-occipital (P3, P4, Pz, O1, O2) ROIs.** From left to right increasing WM-load levels (low, medium, and high). The black horizontal lines in the single TFR-plots denote the lower and upper border of the traditional alpha frequency band (i.e., 8 and 13 Hz). Blue colors signal event-related desynchronization (ERD), red colors signal event-related synchronization (ERS) measured in percent with respect to a baseline condition (see Section “Data Preprocessing and Analysis” for a more detailed description of the calculation of the TFR-plots). The small plots in between the larger TFRs indicate statistically significant differences in ERD/ERS%-values between two adjacent load levels (columns) and tasks (rows). Green color indicates statistically non-significant data points, blue and red colors indicate statistically significant ERD/ERS%-values between adjacent TFR-plots (*p* < 0.05).

The TFR plots (**Figure 3**) show the ERD/ERS%-values (i.e., the oscillatory activity) over time for the three tasks and three load levels for the two different ROIs. The TFR plots were calculated in analogy to a previous study investigating the *N*-back task (Pesonen et al., 2007).

**FIGURE 4:**
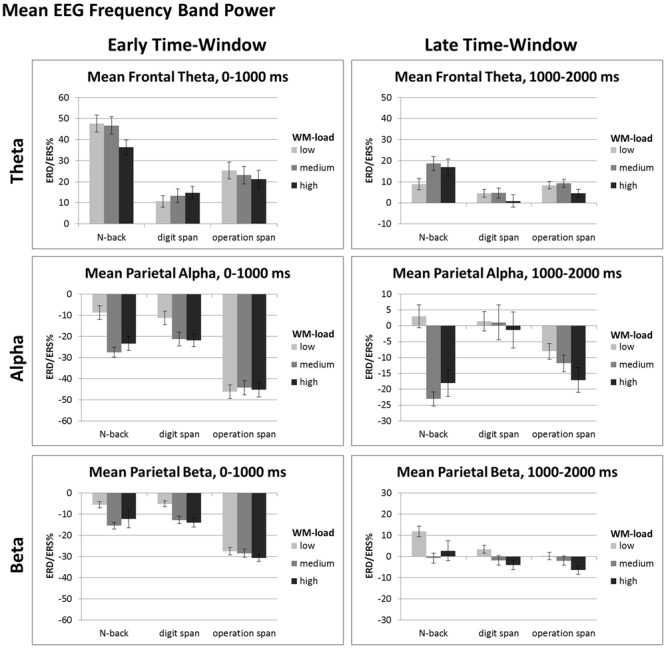
**Mean ERD/ERS%-values of the early (i.e., 0–1000 ms post-stimulus onset) and late (i.e., 1000–2000 ms post-stimulus onset) data analysis window for mean frontal-central theta (4–6 Hz), mean parietal-occipital alpha (8–13 Hz), and mean parietal-occipital beta (14–24 Hz) EEG frequency band power of the *N*-back, Dspan, and Ospan task, and the WM-load levels low, medium, and high.** Black error bars indicate ±1 standard error of the mean.

Our study extends Pesonen et al. (2007) by examining alpha, beta, and theta ERD/ERS% not only for the *N*-back task but also for WM span tasks. Most important, the alpha and beta effects of the *N*-back TFRs in the current study replicate the results reported by Pesonen et al. (2007) as well as other studies analyzing TFRs of the *N*-back task (Krause et al., 2010; Palomäki et al., 2012; Scharinger et al., 2015), thus confirming the validity of the current results. Generally (see **Figure 3**), we observed the typical topographical distribution of TFR effects: For all tasks the alpha and beta frequency band power effects were maximally over parietal electrodes (Pesonen et al., 2007; Krause et al., 2010; Palomäki et al., 2012; Scharinger et al., 2015). However, the frequency band power effects were in general widely spread over the scalp, that is, an alpha or beta ERD could be also observed at frontal electrodes (and theta effects at parietal electrodes), yet being of a decreased magnitude at this location. In sum, most EEG frequency band power effects could be comparably observed at the different ROIs we inspected. Therefore, in line with literature (e.g., Gevins and Smith, 2000), we restricted our more comprehensive statistical analyses of the mean ERD/ERS% data to the classical recording sites (i.e., frontal-central electrodes for the theta and parietal-occipital electrodes for the alpha and beta frequency band power). The ANOVAs were based on the averaged ERD/ERS%-values that are given in **Figure 4**.

#### Alpha Frequency Band Power Effects

Alpha ERD effects could be observed in all tasks in the traditional frequency band range (i.e., between 8 and 13 Hz, see black horizontal lines in the TFR-plots in **Figure 3**), yet a significant load-effect in terms of a significant decrease of the alpha power for increasing load-levels could only be observed in the *N*-back and the Dspan task (for details, see below). The three-factorial repeated measures ANOVA that we run on the mean EEG alpha power data at parietal-occipital recording sites revealed a main effect of task, *F*(2,38) = 11.56, *p* = 0.001, $\eta_{p}^{2}$ = 0.38, time-window, *F*(1,19) = 46.49, *p* < 0.001, $\eta_{p}^{2}$ = 0.71, and load, *F*(2,38) = 12.24, *p* = 0.001, $\eta_{p}^{2}$ = 0.39, as well as two-way interactions between task and time-window, *F*(2,38) = 36.66, *p* < 0.001, $\eta_{p}^{2}$ = 0.66, task and load, *F*(4,76) = 10.24, *p* < 0.001, $\eta_{p}^{2}$ = 0.35, and a three-way interaction between task, time-window, and load, *F*(4,76) = 6.30, *p* = 0.002, $\eta_{p}^{2}$ = 0.25. To resolve this three-way interaction, we ran two additional two-factorial repeated-measures ANOVAs, one for each time-window.

For the early time-window (0–1000 ms post-stimulus onset, see **Figure 4**, left) the ANOVA revealed a main effect of task, *F*(2,38) = 26.81, *p* < 0.001, $\eta_{p}^{2}$ = 0.59 and a main effect of load, *F*(2,38) = 15.53, *p* < 0.001, $\eta_{p}^{2}$ = 0.45, that were qualified by an interaction between task and load, *F*(4,76) = 11.52, *p* < 0.001, $\eta_{p}^{2}$ = 0.38. In the *N*-back task as well as in the Dspan task the alpha ERD increased significantly between the low-load and the medium-load level (*N*-back, low: -5.63%, medium: -24.44%, *p* < 0.001; Dspan, low: -8.20%, medium: -18.23%, *p* = 0.007). In both tasks, the alpha ERD of the medium and the high-load level (*N*-back, high: -20.29%, Dspan, high: -18.78%) were statistically not different (both *p* > 0.519), that is, we did not observe any further increase of the alpha ERD (as can be seen in **Figure 4** in the *N*-back task numerically we even observed a slight decrease of the alpha ERD for the high-load level, i.e., the 3-back condition, as compared to the medium-load level, i.e., the 2-back condition). In contrast, in the Ospan task we did not observe any significant increase of the alpha ERD in the early time-window for increased WM-load (low-load: -43.10%, medium: -41.23%, high: -42.23%, all *p* > 0.786). On all three load levels the Ospan task showed an overall more pronounced alpha ERD as compared to the *N*-back and the Dspan task (all *p* ≤ 0.001). On all load-levels the alpha ERDs of the *N*-back task and the Dspan task were statistically not different (all *p* ≥ 0.457).

In the late time-window (i.e., 1000–2000 ms post-stimulus onset, see **Figure 4**, right side), the overall result pattern was comparable to the result pattern of the early time-window, yet some differences did occur. In general, the alpha ERD was less pronounced in this latter time-window as compared to the early time-window (see main effect of time-window reported above). As in the early time-window, the two-factorial repeated-measures ANOVA revealed a main effect of task, *F*(2,38) = 4.97, *p* = 0.025, $\eta_{p}^{2}$ = 0.21, a main effect of load, *F*(2,38) = 9.23, *p* = 0.003, $\eta_{p}^{2}$ = 0.33, and an interaction between these two factors, *F*(4,76) = 8.19, *p* < 0.001, $\eta_{p}^{2}$ = 0.30. In this late time-window, the Dspan task did not show any significant increase of the alpha ERD for increasing load levels (low: 4.52%, medium: 4.149%, high: 1.78%, all *p* = 1.00). Noteworthy, as indicated by the positive ERD/ERS%-values, we observed a global alpha ERS effect in the Dspan task in the late time-window. In contrast to the Dspan task, the *N*-back task showed increasing alpha ERDs for increased WM-load in this time-window. The increase in alpha ERD, like in the early time-window, was significant only between the low-load (6.05%) and the medium-load level (-19.97%, *p* < 0.001), but not between the medium-load and the high-load level (-14.99%, *p* = 0.551). The Ospan task numerically showed increasing alpha ERDs for increasing load levels (low: -4.94%, medium: -8.72%, high: -13.99%), however, none of the differences reached statistical significance (all *p* ≥ 0.161). On the low-load level, the alpha ERD of the *N*-back task and the Dspan task were statistically not different (*p* = 1.00). As in the early time-window, the Ospan task showed a more pronounced alpha ERD as the *N*-back and the Dspan task, however, this time only in the low-load condition (all *p* ≤ 0.007). In the medium-load condition, the Ospan showed a significantly more pronounced alpha ERD as compared to the *N*-back task (*p* = 0.013), and a numerically more pronounced alpha ERD as the Dspan task, yet this difference being not significant (*p* = 0.231). The alpha ERD of the *N*-back task was more pronounced as compared to the alpha ERD of the Dspan task (*p* = 0.004). In the high-load condition, the alpha ERD of the *N*-back and the Ospan task numerically were more pronounced as the alpha ERD of the Dspan task, yet statistically this difference was not significant (all *p* ≥ 0.122).

In sum, the following findings of the alpha ERD are noteworthy: In the early time-window, the Ospan task showed no significant load-effect and an overall more pronounced alpha ERD as compared to the *N*-back task and the Dspan task. The *N*-back and the Dspan task showed a comparable result pattern with respect to the alpha ERD in the early time-window. In contrast, in the late time-window the Dspan task did not show any load-effect. Furthermore, the Dspan task showed overall positive alpha ERD values (i.e., an alpha ERS). The *N*-back task in contrast showed an increased alpha ERD for increased load levels (yet significantly only between low-load and medium-load). The Ospan only numerically showed an increased alpha ERD for increased load in the late time-window.

The result pattern of the Ospan task showing overall more pronounced alpha ERD as compared to both, the *N*-back and the Dspan as well as showing no significantly increased alpha ERD for increasing load-levels, indicated that the Ospan in general might have been the most demanding task for the participants. We conducted an additional statistical analysis that considered in the Ospan task only the EEG data epochs of those trials of which the digits were later on correctly recalled. In doing so, we sought to minimize potential effects of WM over-load that might have occurred in some subjects in this task and that might have masked the load-effect. Because of this rather strict criterion five participants were excluded from the EEG data analysis as for them in the high-load condition of the Ospan too few data epochs (<15) of correctly recalled digits remained for analysis. On the remaining 15 subjects, we conducted the same ANOVAs as reported above. In general, we observed comparable effects, yet for the late time-window the alpha ERD in the Ospan did now show not only numerically but also statistically significant increased alpha ERD-values for increased WM load, as expected (low: -6.15% to medium: -13.76%, *p* = 0.045, medium to high: -25.13%, *p* < 0.001)^3^.

#### Beta Frequency Band Power Effects

The result-pattern of the beta frequency band power was comparable to the alpha ERD effects reported above, however, some important differences did occur. The three-factorial repeated measures ANOVA conducted on the mean beta power data at parietal-occipital electrodes revealed a main effect of load, *F*(2,38) = 10.64, *p* = 0.002, $\eta_{p}^{2}$ = 0.40, task, *F*(2,38) = 12.98, *p* < 0.001, $\eta_{p}^{2}$ = 0.41, and time-window, *F*(1,19) = 161.38, *p* < 0.001, $\eta_{p}^{2}$ = 0.90, with the latter two qualified by a significant interaction between time-window and task, *F*(2,38) = 28.00, *p* < 0.001, $\eta_{p}^{2}$ = 0.60. The three-way interaction between task, time-window, and load was only marginally significant *F*(4,76) = 2.36, *p* = 0.061, $\eta_{p}^{2}$ = 0.11.

Overall the beta ERD increased for increasing WM-load (low: -2.50%, medium: -8.93%, high: -9.50%), yet significantly only between low and medium respective high-load (all *p* ≤ 0.012) and not between medium and high-load (*p* = 1.00). For all tasks, the beta ERD was overall more pronounced in the early as compared to the late time-window (early: -15.61%, late: 1.65%, all *p* < 0.001). In the early time-window, the beta ERD of the Ospan (-27.60%) was overall more pronounced as compared to both, the *N*-back (-9.85%) and the Dspan (-9.40%, all *p* < 0.001) with the latter two showing no statistical differences (*p* = 1.00). In the late time-window, the beta ERD of all three tasks was comparably pronounced (*N*-back: 5.91%, Dspan: 0.50%, Ospan: -1.46%, all *p* > 0.124).

In sum, the beta ERD showed a comparable result pattern as the alpha ERD yet with more global load-effects. For increasing WM load the beta ERD was more pronounced, most pronounced between low-load and medium-load. The beta ERD of the *N*-back and the Dspan was comparably pronounced with the Ospan showing a larger beta ERD in the early time-window as compared to both, the Dspan and the *N*-back task.

#### Theta Frequency Band Power Effects

The three-factorial repeated-measures ANOVA of the mean theta frequency band power at frontal-central electrodes revealed a main effect of task, *F*(2,38) = 14.21, *p* < 0.001, $\eta_{p}^{2}$ = 0.43, time-window, *F*(1,19) = 57.59, *p* < 0.001, $\eta_{p}^{2}$ = 0.75, and a main effect of load, *F*(2,38) = 4.58, *p* = 0.017, $\eta_{p}^{2}$ = 0.19. These main effects were qualified by a significant interaction between task and time-window, *F*(2,38) = 10.29, *p* < 0.001, $\eta_{p}^{2}$ = 0.35, time-window and load, *F*(2,38) = 3.76, *p* = 0.032, $\eta_{p}^{2}$ = 0.17, and a three-way interaction between time-window, task, and load, *F*(4,76) = 12.16, *p* = 0.001, $\eta_{p}^{2}$ = 0.39. To resolve the three-way interaction, we run two additional two-factorial ANOVAs, one for each time-window.

For the early time-window (cf. **Figure 4**, left part), the ANOVA revealed a main effect of task, *F*(2,38) = 15.67, *p* < 0.001, $\eta_{p}^{2}$ = 0.45, and a main effect of load, *F*(2,38) = 4.88, *p* = 0.013, $\eta_{p}^{2}$ = 0.20, that were qualified by a significant interaction, *F*(2,76) = 5.73, *p* < 0.001, $\eta_{p}^{2}$ = 0.23. On all load-levels, the theta ERS was most pronounced in the *N*-back task (low: 47.51%, medium: 46.59%, high: 36.17%) as compared to both, the Dspan task (low: 10.60%, medium: 13.14%, high: 14.62%) and the Ospan task (low: 25.32%, medium: 22.99%, high: 21.18%, all *p* ≤ 0.039). For the medium and the high-load level the theta ERS of the Dspan and the Ospan were statistically not different (all *p* ≥ 0.333), yet for the low-load level the theta ERS of the Ospan was more pronounced as the theta ERS of the Dspan task (*p* = 0.043). With respect to the load-effects, the theta ERS of the high load level was significantly reduced as compared to both, the low and the medium load level (both *p* ≤ 0.003), with the latter two showing no significant differences (*p* = 1.00). This result might indicate the generally demanding *N*-back task that led to some WM-overload under the high load level as indicated by the decreasing theta power. Both, the Dspan and the Ospan did not show any significant load-effect in the early time-window (all *p* ≥ 0.153).

In the late time-window (cf. **Figure 4**, right part), we observed main effects of task, *F*(2,38) = 6.45, *p* = 0.004, $\eta_{p}^{2}$ = 0.25, and load, *F*(2,38) = 3.80, *p* = 0.031, $\eta_{p}^{2}$ = 0.17, that were qualified by a significant interaction between task and load, *F*(4,76) = 5.21, *p* = 0.001, $\eta_{p}^{2}$ = 0.22. Only the *N*-back task showed a significantly increased theta ERS between the low (8.75%) and the medium-load level (18.48%, *p* = 0.009). Between the medium-load and the high-load level the theta ERS decreased slightly, but not significantly (high-load: 16.78%, *p* = 1.00). In the Ospan task the theta ERS increased slightly for the medium-load (9.17%) as compared to the low-load (8.21%), yet this difference was not significant (*p* = 1.00). Comparably to the *N*-back task in the Ospan task the theta ERS decreased for the high-load level (4.45%) as compared to the medium-load (*p* = 0.034) and, yet only marginally significant, low-load (*p* = 0.074). The Dspan task did not show any significant load-related effect of the theta ERS (low: 4.34%, medium: 4.51%, high: 0.71%, all *p* ≥ 0.320). The theta ERS was comparably pronounced for all three tasks on the low high-load level (cf. **Figure 4**; low-load: all *p* > 0.264). On the medium-load level, the theta ERS of the *N*-back task was more pronounced as compared to the Dspan task (*p* = 0.008), and numerically also as compared to the Ospan task (yet only marginally significant, *p* = 0.075). The theta ERS of the Dspan task and the Ospan task were statistically not different on the medium-load level (*p* = 0.223). Comparably, for the high-load level the theta ERS of the *N*-back task was more pronounced as compared to the Dspan task (*p* = 0.026), and numerically also as compared to the Ospan task (yet only marginally significant, *p* = 0.070), with the theta ERS of the Dspan task and the Ospan task being statistically not different (*p* = 0.828).

To sum up, with respect to the load-related theta ERS, only the *N*-back task showed an increased theta ERS for medium-load as compared to the low-load level, but only in the late time-window. In the early time-window, the overall theta ERS of the *N*-back task was more pronounced as compared to both, the Dspan task and the Ospan task. In the late time-window, this difference was qualified by an interaction between task and WM-load.

### Correlational Data Analysis

The results of the exploratory correlational data analysis are given in **Tables 1**–**4**. In line with literature (e.g., Redick and Lindsey, 2013), the behavioral performance scores of all three tasks correlated positively with each other (see **Table 1**). Also the ERD/ERS%-values of different load-levels within single tasks correlated positively with each other (see **Tables 2**–**4**). More interestingly, however, in both, the Ospan (see **Table 2**) as well as the *N*-back (see **Table 4**) we observed a negative correlation between behavioral performance and the parietal alpha ERD (especially for the higher load-levels). The higher the performance of the subjects in the three tasks, the more pronounced were the alpha ERDs in the *N*-back and the Ospan task (note that an increased alpha ERD is characterized by more negative values). A comparable result-pattern could be observed in these tasks for task performance and the parietal beta ERD. Importantly, in the Dspan task we observed no correlation between behavioral task performance and alpha (or beta) ERD (see **Table 3**). Behavioral task performance did not correlate significantly with frontal theta ERS in any of the tasks (see **Tables 2**–**4**).

**Table 1:** Pearsons’ correlations coefficients (two-tailed): behavioral task performance.

Variable	1	2	*M*	*SD*	*N*
**Behavioral Performance**					
1. Ospan Score			0.64	0.26	20
2. Dspan Score	0.73^∗∗∗^		0.73	0.14	20
3. *N*-back Score	0.43^+^	0.53^∗^	0.82	0.06	20

*^+^*p* < 0.10, ^∗^*p* ≤ 0.05, ^∗∗^*p* ≤ 0.01, ^∗∗∗^*p* ≤ 0.001.*

**Table 2:** Pearsons’ correlations coefficients (two-tailed): Ospan task behavioral performance and EEG ERD/ERS%-values.

Variable	1	2	3	*M*	*SD*	*N*
**Behavioral Performance**						
1. Ospan score				0.64	0.26	20
**EEG frontal theta ERS**						
2. Ospan, low-load	0.17			16.77	14.14	20
3. Ospan, medium-load	0.11	0.90^∗∗∗^		16.08	13.22	20
4. Ospan, high-load	0.10	0.91^∗∗∗^	0.91^∗∗∗^	12.81	15.67	20
**EEG parietal alpha ERD**						
2. Ospan, low-load	-0.41^+^			0.21	12.90	20
3. Ospan, medium-load	-0.50^∗^	0.94^∗∗∗^		-22.20	19.53	20
4. Ospan, high-load	-0.66^∗∗∗^	0.88^∗∗∗^	0.95^∗∗∗^	-17.64	24.43	20
**EEG parietal beta ERD**						
2. Ospan, low-load	-0.39^+^			4.40	9.29	20
3. Ospan, medium-load	-0.43^+^	0.72^∗∗∗^		-6.81	12.81	20
4. Ospan, high-load	-0.64^∗∗^	0.76^∗∗∗^	0.90^∗∗∗^	-3.50	24.03	20

*^+^*p* < 0.10, ^∗^*p* ≤ 0.05, ^∗∗^*p* ≤ 0.01, ^∗∗∗^*p* ≤ 0.001.*

**Table 3:** Pearsons’ correlations coefficients (two-tailed): Dspan task behavioral performance and EEG ERD/ERS%-values.

Variable	1	2	3	*M*	*SD*	*N*
**Behavioral Performance**						
1. Dspan score				0.73	0.14	20
**EEG frontal theta ERS**						
2. Dspan, low-load	0.31			7.47	9.44	20
3. Dspan, medium-load	0.07	0.82^∗∗∗^		8.83	12.24	20
4. Dspan, high-load	-0.07	0.81^∗∗∗^	0.93^∗∗∗^	7.67	15.51	20
**EEG parietal alpha ERD**						
2. Dspan, low-load	0.08			-1.84	10.40	20
3. Dspan, medium-load	0.11	0.76^∗∗∗^		-7.07	20.62	20
4. Dspan, high-load	-0.08	0.69^∗∗∗^	0.93^∗∗∗^	-8.50	23.99	20
**EEG parietal beta ERD**						
2. Dspan, low-load	0.05			0.45	6.25	20
3. Dspan, medium-load	0.11	0.91^∗∗∗^		-6.02	9.69	20
4. Dspan, high-load	-0.06	0.76^∗∗∗^	0.89^∗∗∗^	-7.72	13.42	20

*^+^*p* < 0.10, ^∗^*p* ≤ 0.05, ^∗∗^*p* ≤ 0.01, ^∗∗∗^*p* ≤ 0.001.*

**Table 4:** Pearsons’ correlations coefficients (two-tailed): *N*-back task behavioral performance and EEG ERD/ERS%-values.

Variable	1	2	3	*M*	*SD*	*N*
**Behavioral Performance**						
1. *N*-back score				0.82	0.06	20
**EEG frontal theta ERS**						
2. *N*-back, low-load	0.14			28.13	12.94	20
3. *N*-back, medium-load	0.29	0.78^∗∗∗^		32.54	17.44	20
4. *N*-back, high-load	0.26	0.76^∗∗∗^	0.84^∗∗∗^	26.47	17.79	20
**EEG parietal alpha ERD**						
2. *N*-back, low-load	-0.36			0.21	12.90	20
3. *N*-back, medium-load	-0.43^+^	0.78^∗∗∗^		-22.20	19.53	20
4. *N*-back, high-load	-0.68^∗∗∗^	0.57^∗∗^	0.83^∗∗∗^	-17.64	24.43	20
**EEG parietal beta ERD**						
2. *N*-back, low-load	-0.30			4.40	9.29	20
3. *N*-back, medium-load	-0.18	0.74^∗∗∗^		-6.81	12.81	20
4. *N*-back, high-load	-0.16	0.29	0.59^∗∗∗^	-3.50	24.03	20

*^+^*p* < 0.10, ^∗^*p* ≤ 0.05, ^∗∗^*p* ≤ 0.01, ^∗∗∗^*p* ≤ 0.001.*

### P300 Amplitude

**FIGURE 5:**
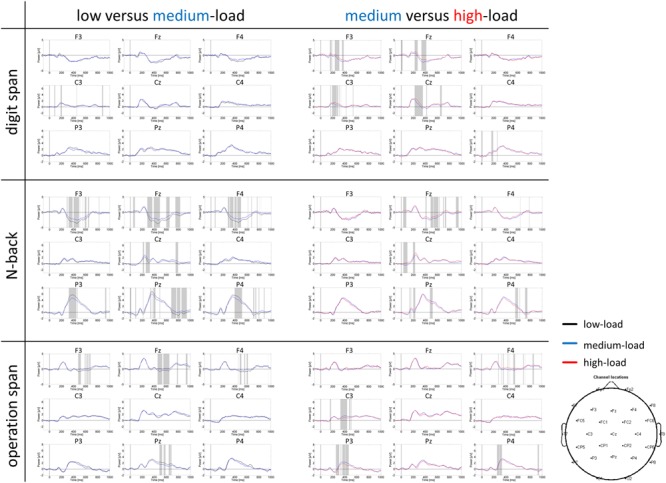
**Grand average ERP curves at frontal (F3, Fz, F4), central (C3, Cz, C4), and parietal (P3, Pz, P4) electrodes for the three tasks and three load levels.** Left part of the figure compares the low and medium-load categories, right part the medium and high-load categories. Paired sampled *t*-tests (two-tailed) were conducted between load levels for each time point (EEGLAB bootstrapping statistics, using false-discovery-rate corrections for multiple comparisons). Gray shaded areas indicate significantly different data points (*p* < 0.01).

**FIGURE 6:**
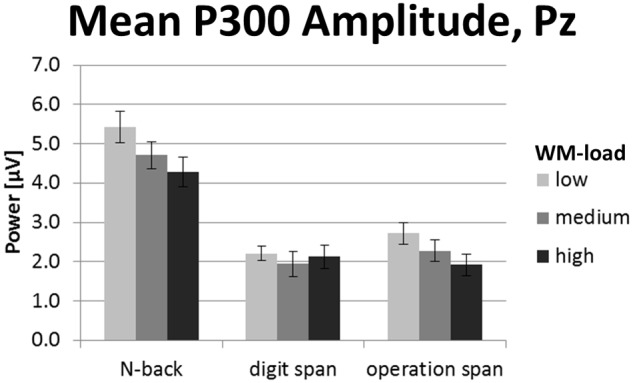
**Mean P300 amplitude (300–500 ms post-stimulus onset) at electrode Pz of the *N*-back, Dspan, and Ospan task, and the WM-load levels low, medium, and high.** Black error bars indicate ±1 standard error of the mean.

Grand average ERP curves for the three tasks and three load levels are shown in **Figure 5** for frontal, central, and parietal electrode locations. Importantly, we restricted our interpretation of the ERP data to the mean P300 amplitude at parietal electrodes that is given in **Figure 6**. This was because the P300 has been analyzed previously in the *N*-back task at these electrode positions (McEvoy et al., 2001; Watter et al., 2001; Chen et al., 2008; Scharinger et al., 2015). As can be seen in **Figure 5**, in the *N*-back task we observed a strong positive deflection that was maximally over parietal electrodes between 300 and 500 ms. In the Ospan task and the Dspan task this positive deflection is far less visible.

The two-factorial repeated-measures ANOVA conducted on the mean P300 amplitude data (300–500 ms post-stimulus onset) at electrode Pz revealed a main effect of task, *F*(2,38) = 33.84, *p* < 0.001, $\eta_{p}^{2}$ = 0.64, as well as a main effect of load, *F*(2,38) = 9.07, *p* = 0.001, $\eta_{p}^{2}$ = 0.32. We did not observe a significant interaction between task and load for P300 amplitude, *F*(4,76) = 2.05, *p* = 0.096, $\eta_{p}^{2}$ = 0.10. The *N*-back showed a significantly more pronounced P300 amplitude (4.81 μV) as compared to both span tasks (Dspan: 2.09 μV, Ospan, 2.31 μV, *p* < 0.001), with the latter two showing no statistically significant difference (*p* = 1.00). Overall, the P300 amplitude decreased for increased WM-load levels (low-load: 3.45 μV, medium-load: 2.98 μV, high-load: 2.78 μV), showing a significant difference between the low-load level and the high-load level (*p* = 0.004), a marginally significant difference between the low-load and the medium-load level (*p* = 0.051), and no significant difference between medium-load and high-load (*p* = 0.472).

In sum, despite the less pronounced P300 for the span tasks as compared to the *N*-back task, the P300 amplitude overall decreased for increasing WM-load levels. As there was no clear P300 peak detectable in the span tasks, we restricted our analysis of the P300 to the mean amplitude defined as average power in a time-window between 300 and 500 ms post-stimulus onset. Any further analysis of, for example, P300 latency does not appear justified given the P300 shape in the current study.

## Discussion

In the current study, we analyzed EEG correlates of WM processing-load for *N*-back and WM span tasks. To date, these measures, while being extensively reported for *N*-back tasks (e.g., Pesonen et al., 2007; Krause et al., 2010; Palomäki et al., 2012; Scharinger et al., 2015), have rarely (though not all) been used in the context of WM span tasks (see sections “EEG Frequency Band Power Correlates of WM Processing-Load” and “EEG P300 Amplitude Signaling WM Processing Demands”). Especially, the current study is the first one in which the same participants conducted an *N*-back, a complex Ospan, and a simple Dspan task while EEG data were recorded. This allowed us to compare the typical load-related EEG measures (i.e., the theta, alpha, and beta frequency band power and the P300 amplitude) in comparison for the different tasks. Overall, we expected these measures to show more WM processing-load in the *N*-back and the Ospan task as compared to the Dspan task, as conceptually the *N*-back and the Ospan task share more commonalities with respect to WM processing (i.e., the EFs updating, shifting, and inhibition), as compared to the simple Dspan task (see Conceptual Task Analyses of WM Span Tasks and N-Back Tasks).

Briefly summarized, the current data only partly support current theoretical accounts on these WM tasks. Both, the *N*-back and the Ospan task showed an alpha ERD that was prolonged in time for higher WM-load levels, whereas the Dspan task did not show such a temporal prolongation of the alpha ERD effects. However, with respect to the magnitude of the alpha (and beta) ERD effects (especially in the early time-window), the *N*-back and the Dspan task seemed to be more similar as compared to the Ospan task which showed overall the most pronounced alpha (and beta) ERD effects. In contrast, with respect to the P300 amplitude as well as the theta frequency band power, the *N*-back task differed significantly from the Ospan task and the Dspan task in showing the overall largest P300 amplitude and the most pronounced theta ERS. Despite the larger P300 amplitude for the *N*-back as compared to the span tasks, all tasks showed a decreasing P300 amplitude for increasing WM-load levels. In contrast, the frontal theta ERS did only show a significant load-related effect for the *N*-back task (i.e., an increase in theta power for increased WM-load in the late time-window). In the following, we will discuss the main outcomes in detail.

### EEG Alpha Frequency Band Power

With respect to the frequency band power, theoretical accounts on the three WM-tasks (see Conceptual Task Analyses of WM Span Tasks and N-Back Tasks) predict neuronal activity (and hence, an oscillatory pattern) being more similar between the *N*-back and the Ospan as compared to the Dspan task. As the decrease in EEG alpha power (i.e., the increasing alpha ERD) can be interpreted to reflect demands on WM processing (Gevins et al., 1997; Stipacek et al., 2003; Pesonen et al., 2007; Engel and Fries, 2010), we expected to observe in the *N*-back and the Ospan task more pronounced alpha ERDs than in the Dspan task. These expectations are in line with the common assumption about the functional role of alpha ERD, namely, that the higher the alpha ERD, the more different neuronal networks are active (Klimesch, 1999; Pfurtscheller and Lopes da Silva, 1999). Thus, the alpha ERD might be related to the number of cognitive processes (e.g., EFs) that are necessary for task performance. Generally, the outcomes of the current study are in line with the assumption that varying demands on EFs in the different WM tasks are reflected by the oscillatory activity in the alpha frequency band range (i.e., in the alpha ERD). In the following, we will briefly summarize and discuss the most important findings with respect to this assumption.

First, when taking both, magnitude and timely prolongation in combination into account, the alpha ERD in the Dspan task was overall less pronounced as compared to the alpha ERD in the Ospan and the *N*-back task. Although the demands of the Dspan and the Ospan task on WM storage were comparable in terms of the number and difficulty of the to-be-memorized stimuli, the alpha ERD was more pronounced in the complex Ospan as compared to the simple Dspan task. This may be seen as a strong hint of EEG alpha power reflecting the number of simultaneously required EFs for task performance (and hence WM processing-load) instead of reflecting simple STM storage-load (which is comparable between the two span tasks). In the *N*-back task and the Ospan task, among others, EFs like updating, shifting, and inhibition may be active in the time-window we choose for data analysis. In contrast, in the Dspan task less cognitive processes (i.e., presumably only updating processes) may be active, which may be directly reflected in the strength and timing of the overall alpha ERD.

Noteworthy, however, when taking only the magnitude (without consideration of the timely prolongation) into account (i.e., when focusing on the early time-window), the alpha ERD of the Dspan and the *N*-back task were quite similar and both less pronounced as the alpha ERD of the Ospan task. This outcome is rather unexpected given the conceptual considerations stated above (see Conceptual Task Analyses of WM Span Tasks and N-Back Tasks). However, it is in line with observations in studies focusing on behavioral performance data that often report higher correlations between *N*-back and simple span tasks than between *N*-back and complex span tasks (cf. Redick and Lindsey, 2013; also see the correlational results of the current study, **Table 1**). Thus, the current EEG alpha power data seem to reflect those behavioral outcomes, indicating a strong link between alpha ERD and behavioral performance.

When focusing on the late time-window, both, the *N*-back and the Ospan task, but not the Dspan task, showed an increased alpha ERD for increased WM-load (i.e., timely prolonged alpha ERD effects). This fits with the conceptual considerations that the *N*-back task and the Ospan task demand WM processing more severely as compared to the Dspan task. This interpretation is corroborated further by the observation that in the late time-window the Dspan task overall showed an alpha ERS (as indicated by the positive ERD/ERS%-values) rather than an alpha ERD. Alpha ERS in WM tasks have been previously described for the retention phase of WM content (Jensen et al., 2002; Palva and Palva, 2007). Thus we may conclude that because of the less complex nature of the Dspan task as compared to the other two WM tasks, in this task WM processing (i.e., WM updating) takes place early after stimulus onset (shown by the alpha ERD effect in the early time-window), whereas in the late time-window no further WM processing takes place (i.e., a purely retention period as indicated by the alpha ERS). In contrast, because of their more complex nature and higher demands on WM processing, in the *N*-back and the Ospan task demands on WM processing are raised throughout the overall task and intermixed with any potential retention phase. In sum, the alpha ERD might index the timing of WM processing-load.

### EEG Beta Frequency Band Power

In the beta frequency band, the load-associated effects resembled those of the alpha frequency band (i.e., an increased beta ERD for increased WM-load), albeit showing fewer differences between the tasks. Beta ERD effects for increased load in an *N*-back task paradigm have been consistently observed in several studies (Pesonen et al., 2007; Krause et al., 2010; Palomäki et al., 2012). Although these authors hypothesized the beta ERD effects as being cognitively induced, they could not rule out a purely motor explanation as their *N*-back task required overt motor activity (key-press). Our data show beta ERD effects in all three tasks, even in the Dspan task that required no key-presses, thus underlining the character of the beta ERD as reflecting cognitive (WM) processes (cf. Engel and Fries, 2010).

### EEG Theta Frequency Band Power

The EEG theta power ERS was overall most pronounced in the *N*-back task as compared to both span tasks. Additionally, only in the *N*-back task we observed an increased theta ERS for increased WM-load (in the late time-window). This increased theta ERS for increased WM-load in the *N*-back task is in line with literature (Gevins et al., 1996, 1997; Gevins and Smith, 2000). As described in the Section “Conceptual Task Analysis of the *N*-Back Task,” one important feature of this WM task is that it mainly and simultaneously demands WM processing (i.e., the EFs updating, shifting, and inhibition) and less WM storage as compared to the span tasks. Neuronal oscillatory activity in the theta frequency band range has been hypothesized to especially reflect cognitive control processes in WM (Sauseng et al., 2010). The need for such control processes might be highest in the *N*-back task due to the closely intertwined demands on WM processing (i.e., EFs) in this task as compared to the span tasks. This might result in the overall increased theta ERS in the *N*-back task as compared to the span tasks.

### P300 Amplitude

As for the EEG frequency band power data, we expected to observe changes in the P300 amplitude to be more comparable between the *N*-back and the Ospan task as compared to the Dspan task. The outcome of the P300 amplitude only partly supported the conceptual considerations described in the task analyses (see Conceptual Task Analyses of WM Span Tasks and N-Back Tasks). First, the *N*-back showed an overall more pronounced P300 amplitude as the Dspan and the Ospan task. Second, all tasks showed a decrease in P300 amplitude for increasing WM-load levels (i.e., we did not observe a significant interaction between task and load levels). However, although unexpected, this finding is noteworthy, as it shows that irrespective of the task-related magnitude of the P300 amplitude, increasing WM-load leads to a decreasing P300 amplitude. Thus, the P300 amplitude turned out as a good measure of changes in the overall WM-load, irrespective of the concrete task.

Noteworthy, the overall more pronounced P300 amplitude in the *N*-back task as compared to the span tasks resemble the pattern of the EEG theta power results, showing an overall more pronounced theta ERS for the *N*-back as compared to the span tasks. This is in line with literature reporting increased theta power accompanied with increased P300 amplitude (Spencer and Polich, 1999). Generally, EEG theta power seems to be sensitive to comparable task variables that also influence the P300 like target probability and task difficulty (e.g., Spencer and Polich, 1999; Mazaheri and Picton, 2005), thus indicating a close link between the P300 and the EEG theta frequency band. Thus, the results of the current study showing the pronounced theta and P300 effects for the *N*-back task as compared to the span tasks might underline a close connection between the two measures. However, a more in-depth comparison of the different WM-load measures *per se* was beyond the scope of the current study. Functionally, the observation of a more pronounced P300 and theta power in the *N*-back task as compared to the span tasks might be a strong hint that attentional and cognitive control processes are especially required in this task for task performance (cf. EEG Theta Frequency Band Power).

Importantly, the outstanding character of the *N*-back task in demanding WM processing might be corroborated further by the observation that on all measures we analyzed, the strongest WM load-related change was between low and medium WM-load (i.e., the 1-back and the 2-back load level). For higher load-levels (i.e., the 3-back load-level) a kind of plateau was reached and no further significant change in the electrophysiological measures could be observed. This is noteworthy as in the *N*-back task (especially in the 2-back load level) the WM storage-load is rather low. Thus, the *N*-back might mainly demand WM processing, which might lead to the differences in the EEG theta power and the P300 amplitude of this task as compared to the span tasks.

### Correlational Data Analysis

We examined the correlations between the behavioral performance in the three tasks and the load-related changes in the EEG frequency band power (i.e., the ERD/ERS%-values) of the alpha, beta, and theta frequency bands. In both, the Ospan as well as the *N*-back we observed a quite symmetrical correlational pattern, showing negative correlations between the behavioral performance in these tasks and the alpha ERDs. Such a correlational result-pattern was absent for the Dspan, and also for the frontal theta ERS.

The correlational results are interesting from two perspectives. First, the correlations between the EEG alpha ERD and behavioral performance in the *N*-back and the Ospan mirror our conceptual task analyses (see Conceptual Task Analyses of WM Span Tasks and N-Back Tasks) with respect to dissociable aspects of the tasks. Both, the *N*-back and the Ospan are thought to demand WM processing, whereas, in contrast to that, the Dspan may demand mainly WM storage. Thus, we may assume that the common (and with respect to the Dspan, dissociable) demands on WM could lead to the observed result pattern. Noteworthy, however, this interpretation is rather speculative at this point and clearly requires future research. Second, we did not observe significant correlations for the theta power, which may underline specific functional differences between neuronal activity in the theta frequency band range of the EEG and the alpha (and beta) frequency band (see EEG Frequency Band Power Correlates of WM Processing-Load).

## Conclusion

To sum up, the current study compared typical EEG correlates of WM processing-load like the EEG theta, alpha, and beta frequency band power and the P300 amplitude between a complex Ospan task, a simple Dspan task, and an *N*-back task. These measures have to date been rarely used in WM span tasks to study WM processing-load and a systematic comparison of the three tasks in one study has not been conducted yet. In line with our assumptions concerning commonalities and differences with respect to WM processing-load induced by the *N*-back and the Ospan task as compared to the Dspan task, we found timely more prolonged alpha frequency band power effects in the *N*-back task and the Ospan task, but not in the Dspan task. However, in contrast to assumptions of theoretical accounts defining the Ospan task and the *N*-back task comparably as ‘true’ WM tasks, we also found some differences between these two tasks as well as commonalities between the *N*-back and the Dspan task. Given the results of the P300 and theta frequency band power, the *N*-back task seem to outstandingly demand WM processing and cognitive control as compared to the span tasks. Taken together, the current study pointed out a certain gap between theoretical WM accounts and neurophysiological outcomes when studying different WM tasks like the *N*-back, the Ospan, and the Dspan task in direct comparison using neurophysiological measures, opening the stage for further research.

## Author Contributions

The authors contributed to the work as described in the following. Conception and design of the work: CS and PG. Analysis and interpretation of data for the work: CS, AS, TS, and PG. Drafting the work: CS, AS, TS, and PG. Final approval: CS, AS, TS, and PG. All authors agree to be accountable for all aspects of the work.

## Conflict of Interest Statement

The authors declare that the research was conducted in the absence of any commercial or financial relationships that could be construed as a potential conflict of interest.

## Footnotes

1 Data of the different *N*-back conditions (value, position, form) were recorded to address a second research question, namely the comparison of EEG correlates for different kinds of *N*-back tasks. However, in the current paper, we restricted our research question on the comparison of *N*-back and span tasks. As we used verbal variants of the span tasks (Ospan and Dspan), we analyzed only the verbal *N*-back conditions (‘digit value’). Importantly, in the span tasks, the stimuli were always presented on screen center, whereas in the *N*-back the positions of the stimuli varied within the 3×3 grid. However, as we instructed the participants to always keep fixated the central fixation cross (cf. above), and, furthermore, as the stimuli occurred within the visual field of the participants, we do not expect the stimulus arrangement to affect subjects’ gaze behavior. Furthermore, participants should have fixated the center of the screen in order to use the best perceptual strategy to avoid missing stimuli: Because of the rather short visibility of the stimuli (i.e., 500 ms) and the fact that the stimuli could occur randomly at one of eight different screen positions, keeping a central point fixated would be the best strategy to cover the whole visual field where stimuli could occur at a glance, that is, to avoid missing stimuli.

2 We would like to thank the reviewer for the valuable suggestion to additionally examine the relationship between behavioral performance measures and EEG outcomes.

3 Based on this result, we additionally conducted all statistical data analyses for the selected *N* = 15 subjects. However, as only for the alpha ERD of the Ospan task this led to important statistical differences as compared to the data analyses based on *N* = 20, we decided, for reasons of space, to only report the latter results (i.e., *N* = 20 subjects).
